# Functional connectivity patterns in preschool children associated with working memory performance and digital device use

**DOI:** 10.1038/s41598-025-33555-w

**Published:** 2025-12-30

**Authors:** Nadezhda V. Sutormina, Inna A. Kalabina, Victoria L. Efimova, Elena I. Nikolaeva

**Affiliations:** Department of the Developmental Psychology and Family Pedagogics, Herzen State Pedagogical University, Saint-Petersburg, 191186 Russia

**Keywords:** Functional connectivity, EEG, Preschool children, Working memory, Digital device use, Graph theory, Neuroscience, Psychology, Psychology

## Abstract

Rapid growth in touchscreen technology has made digital devices ubiquitous in early childhood, yet their neurocognitive impact remains unclear. We recorded resting‑state EEG from 31 typically developing 5‑ to 7‑year‑olds immediately before and after a short smartphone‑game session to examine whether brief screen exposure alters functional connectivity patterns in EEG space and how these changes relate to working‑memory performance. Functional connectivity graph‑theoretical metrics (global and local efficiency, clustering coefficient, assortativity and node strength) in delta and beta bands was quantified. Short-term smartphone gameplay did not induce measurable changes in EEG functional connectivity. Cluster analysis identified distinct connectivity patterns across frequency bands. Superior working-memory performance was linked to a more locally segregated and less globally integrated delta-band network. In the beta range, higher-performing children also exhibited greater local clustering, aligning with developmental increases in beta-band segregation. Accordingly, our findings suggest that EEG connectivity metrics can indeed reflect individual differences in working-memory capacity.

**Supplementary Information:**

The online version contains supplementary material available at 10.1038/s41598-025-33555-w.

## Introduction

Over the past two decades, children’s access to technology has expanded dramatically, driven by the proliferation of wireless internet, portable devices, and their increasing accessibility^1^. This surge in technology use among children has become a significant concern for parents and educators, altering daily family dynamics, exacerbating social disparities in gadget utilization and its impact, and potentially limiting opportunities for a healthy lifestyle^2^.

In this context, a notable comparative study conducted in the United States is of particular interest. It investigated two cohorts of children, one growing up in 1997 (*N* = 2193) and another between 2014 and 2016 (*N* = 1009). The data were extracted from the Panel Study of Income Dynamics - Child Development Supplement. Utilizing multivariate regression models, the study assessed total technology screen time, physical activity, unstructured play, and sleep. Results indicated that the total time children spent using digital technology increased by 32% in early childhood and by 23% in middle childhood compared to the 1997 cohort. Furthermore, technology use was inversely related to parental education level, being lowest among children whose parents possessed the highest educational attainment. In the more recent cohort, digital technology engagement correlated with reduced physical activity during middle childhood yet was associated with increased unstructured play in early childhood and more sleep in middle childhood. The findings suggest that the proliferation in screen time among young children has contributed to a reorganization of daily family life. Moreover, these changes have potential implications for children’s health, as well as their cognitive and emotional development^3^.

Equally important findings have been obtained from studies investigating cognitive abilities in individuals who commenced gaming in childhood during the 1980s and have continued into adulthood. Excessive engagement in gaming has been associated with attention deficits, challenges in social interaction, and an elevated risk of obesity. Conversely, certain gamers have exhibited cognitive advantages relative to the average non‑gaming participant. These advantages include a high capacity for visual information processing, better spatial visualization, faster reaction times to external stimuli, and enhanced ability to mentally rotate objects. Research further indicates that when video game engagement is limited to no more than one hour per day, approximately four days a week over a six‑month period, observable improvements can occur in visual attention (the ability to detect and process visual input), spatial attention, and multitasking capabilities^4^.

The contradictory findings from the studies underscore that the impact of digital device use on children’s cognitive development remains highly controversial^1^. Despite growing criticism from the opponents of early childhood screen exposure, these concerns have exerted minimal influence on actual family practices, as the use of digital devices continues to increase. From this vantage point, it is particularly crucial to evaluate both the impact and the quality of a child’s device usage through objective measures, ideally those that, to some extent, reflect brain maturation.

To objectively characterize brain network organization, functional connectivity analysis is a widely employed approach, as it reflects the integration and segregation of neural networks^5–8^. Functional connectivity can be quantified using graph theory, in which networks are modelled as nodes and edges. Nodes typically represent brain regions (e.g., voxels, anatomical areas, or electrodes), while edges correspond to statistical relationships (e.g., correlations) between the time series of activity from those regions. This framework enables the calculation of various metrics that capture distinct aspects of network architecture^9^.

Functional integration denotes the capacity of a network to rapidly link multiple distinct regions or modules. This is frequently quantified by path length: the shorter the average path between nodes indicates the greater the degree of functional integration. Global efficiency, a commonly used metric, reflects the average inverse shortest path length between all pairs of nodes (in the present study, electrodes or sensors)^10^. High global efficiency indicates that information can be quickly and effectively exchanged across disparate network regions, thereby encapsulating the system’s overall capacity for functional integration. Another integration-related metric is the node strength, defined as the sum of the weights of all edges connected to a given node^11^. This measure reflects the overall connectivity of a node within the network and indicates its contribution to global network integration.

Conversely, functional segregation refers to the extent to which a network can be divided into distinct clusters or modules. It can be characterized by the clustering coefficient which quantifies the proportion of existing connections among a node’s neighbors relative to the total number of possible connections between them. Higher clustering coefficient values indicate that neighboring nodes form tightly interconnected clusters, reflecting efficient local information exchange^11^. Another common measure of segregation is modularity, which quantifies how well a network can be partitioned into relatively autonomous communities, groups of nodes that exhibit dense internal interconnections but sparse connections with the remainder of the network^12^.

Additionally, local efficiency quantifies the effectiveness of communication among the neighbors of a given node when that node is removed. This metric provides insights into the resilience of local connections and the redundancy of the network’s local structure. Elevated local efficiency implies robust local information processing and enhanced segregation at the neighborhood level^13^. Assortativity, another pivotal network metric, characterizes the propensity of nodes with similar degree (i.e., number of connections) to interconnect^14^. In assortative networks, highly connected nodes tend to link with other highly connected nodes, resulting in positive assortativity values. These networks are generally considered to be more stable and organized. In contrast, disassortative networks, exhibiting negative assortativity, feature a structure where high‑degree nodes connect more frequently with low‑degree nodes. Such architectures, commonly observed in biological networks, are typically less stable and more susceptible to targeted perturbations^10^. Consequently, assortativity serves as an indicator of network stability and structural robustness^15^. Collectively, global and local efficiency, node strength, clustering coefficient and assortativity provide a comprehensive framework for evaluating the equilibrium among integration, segregation, and stability in functional networks derived from EEG data.

The dynamic interplay between integration and segregation is thought to be optimized within small‑world network architecture. A small‑world network is characterized by high clustering of local nodes and short path lengths between distant nodes, thereby enabling efficient information transmission with minimal wiring cost^16^. Such architecture combines the benefits of both highly modular (segregated) and highly integrated networks. While a highly integrated network supports rapid communication, it also necessitates substantial metabolic and structural resources. Conversely, a highly segregated network may hinder efficient information flow. Small‑world topology addresses this trade‑off by forming densely interconnected clusters that are bridged by hub nodes, thus enabling both specialized processing and global coordination^17^. High segregation indicates the presence of specialized subgroups within the network, whereas substantial integration implies robust global connectivity. The balance between these properties is essential for efficient network function and may vary depending on cognitive state or pathological conditions^14^.

The configuration of brain networks has been shown to be intrinsically linked to cognitive abilities^8^. For instance, in young adults, a more integrated network architecture based on fMRI data correlates with enhanced speed and accuracy in cognitive task performance^18^. In children, fMRI studies have shown that transitions between functional network states occur less frequently, with a single configuration persisting for longer periods^19^. As the brain matures, intermodular connections tend to attenuate, while intramodular connections strengthen, resulting in increased modularity. This growing independence between modules facilitates the functional specialization of distinct brain regions^20^. Furthermore, elevated network modularity has also been proposed as a potential indicator of learning‑related brain plasticity and modular networks may more effectively support the acquisition of novel skills^21^.

Cognitive abilities in children are closely linked to the maturation of brain structures. These abilities can be assessed by evaluating executive functions^22–26^, which mediate the regulation and adaptation of behavior and are critically dependent on the development of the prefrontal cortex, as demonstrated by studies using both animal and human models, including developmental fMRI research in children^27–30^. Among executive functions, inhibitory control and working memory are most frequently evaluated when assessing children’s mental abilities^31^. Inhibitory control denotes the ability to suppress behaviors that are no longer appropriate in a given context^32–34^. It also encompasses the capacity to ignore distracting or irrelevant stimuli during task execution - a faculty that underpins focused attention and goal‑directed behavior^35,36^. Within the framework of executive functions, working memory refers to the temporary storage and manipulation of information necessary to complete multi‑step tasks. It enables individuals to track intermediate stages of a task, anticipate subsequent steps, and work toward completion^37–40^. A large‑scale study of children aged 3 to 19 years documented a nonlinear developmental trajectory of working memory, with two distinct peaks observed in early childhood and adolescence^41^. In contrast, inhibitory control remains comparatively immature during this period, with significant development typically beginning after the age of seven^2^. In the present study, our focus is exclusively on working memory.

These developmental patterns formed the basis for the current study, which aims to evaluate whether the use of digital devices affects 5–7‑year‑old children, by examining functional connectivity parameters and their relationship to working memory. To investigate this question, we first applied principal component analysis (PCA) to reduce the number of variables representing EEG connectivity metrics in the electrode space, such as global and local efficiency, clustering coefficient, assortativity, and node strength derived from adjacency matrices. The resulting two principal components were then subjected to hierarchical cluster analysis to identify potential subgroups of children differing in their EEG connectivity profiles. Finally, we examined whether these subgroups also varied in working-memory performance and in variables related to digital device use.

## Results

Initial exploratory analyses were conducted across multiple EEG frequency bands. However, the sampling adequacy for the alpha and theta ranges was below acceptable thresholds (KMO < 0.5), indicating that these data were not suitable for dimensionality reduction. Therefore, principal component and clustering analyses were performed only for the delta and beta frequency bands, where data quality and sampling adequacy were sufficient. Given the exploratory nature of these analyses, no multiple-comparison correction was applied across EEG bands. In addition, no significant differences in resting-state EEG connectivity metrics were observed before and after smartphone gameplay.

Given the limited sample size, principal component analysis (PCA) was applied separately to the delta and beta bands to reduce data dimensionality prior to clustering. For the delta range, PCA was deemed appropriate based on the results of the Kaiser-Meyer-Olkin (KMO) measure, which yielded a value of 0.531, and Bartlett’s test of sphericity, which was significant (χ² = 200.11, *p* = 0.000). These indices collectively affirmed sufficient sampling adequacy and intercorrelation among variables, justifying the application of PCA. Two principal components (PCs) with the highest levels of explained variance were selected for further analysis (Supplementary Table 1). PC1, accounting for 34% of the variance, captured an integration-segregation gradient within the functional network (Supplementary Table 2). It showed positive loadings on local efficiency, clustering coefficient, and node strength, and negative loadings on global efficiency and assortativity. This pattern suggests that higher PC1 scores correspond to more locally clustered and segregated networks, while lower scores reflect a more globally integrated architecture. PC2 explained 22.8% of the variance and represented a pre-post reconfiguration axis. It loaded positively on post-task global and local efficiency, clustering coefficient, and assortativity, while showing negative loadings for their pre-task counterparts and post-task node strength. This component therefore reflects a transition toward more efficient, locally clustered, and assortatively organized networks after smartphone play, accompanied by a slight reduction in overall connection strength. In the subsequent step, hierarchical cluster analysis was carried out on the two principal components (PC1 and PC2).

**Fig. 1:**
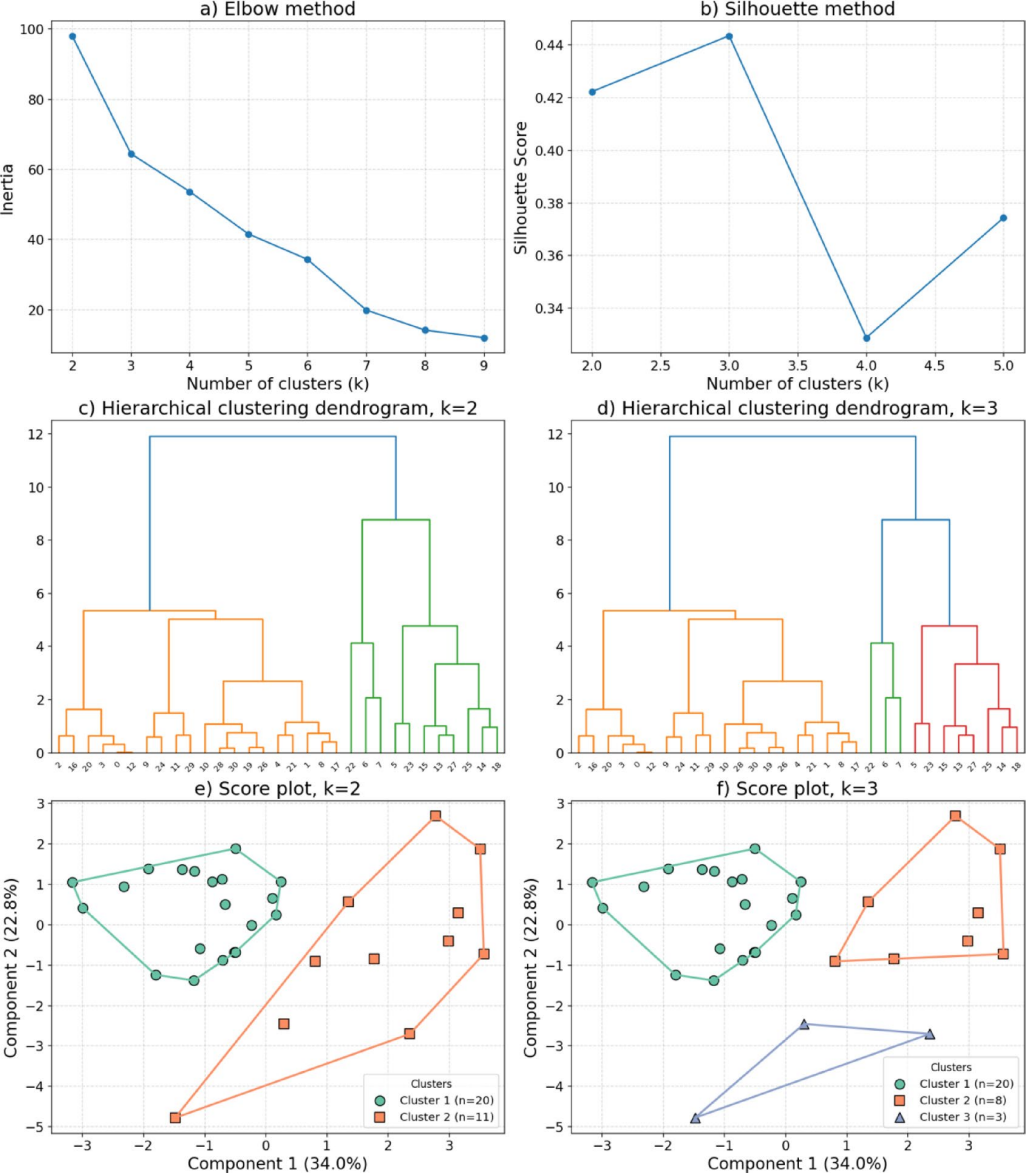
Hierarchical Cluster Analysis results in the delta band (0.5–3.5 Hz). The score plot shows two and three distinct clusters based on the first two principal components.

We examined and compared two- and three-cluster solutions. Figure 1 summarizes the evaluation and selection of the final clustering solution. Panels (a) and (b) show the Elbow and Silhouette results. Both methods suggested that three clusters might provide the best model fit. However, closer inspection revealed that the third cluster included only three participants and showed limited internal stability (Jaccard = 0.49 ± 0.30), indicating that it did not represent a reliable subgroup. According to bootstrap validation (300 iterations, 80% subsampling), the two-cluster solution showed higher and more consistent stability, with Jaccard values of 0.75 ± 0.20 for Cluster 1 and 0.88 ± 0.10 for Cluster 2.

The score plots (e, f) display participants in the space of the first two principal components, each point representing an individual observation projected into the reduced PCA space. In the two-cluster solution (e), clusters are clearly separated: Cluster 1 (green) occupies predominantly negative PC1 values and forms a relatively cohesive group, whereas Cluster 2 (orange) extends across the positive side of PC1 and shows greater internal variability. In the three-cluster solution (f), the third cluster (blue) includes only three participants and appears as a small, isolated group in the lower region of the plot. Taken together, these findings support the use of the two-cluster solution as a more stable and interpretable representation of the data.

The following section presents the differences between the two clusters based on the variables included in the clustering, namely theoretical network metrics of functional connectivity, and those not included in the clustering, such as working memory parameters and parental questionnaire data regarding their own and their children’s digital experience.

**Table 1:** Characteristics of variables included and not included in the clustering analysis in the delta band: differences between the clusters.

Variable	All (*N* = 31)^a^	Cluster 1 (*N* = 20)^a^	Cluster 2 (*N* = 11)^a^	Effect size (95% CI)^b^	*p*-value^c^
Clustering variables					
Global Efficiency (before)	0.53 ± 0.05	0.54 ± 0.03	0.50 ± 0.05	r₍rb₎ = 0.564 (0.209, 0.855)	**0.011**
Local Efficiency (before)	0.55 ± 0.07	0.52 ± 0.06	0.59 ± 0.06	r₍rb₎ = -0.564 (-0.855, -0.191)	**0.011**
Clustering Coefficient (before)	0.36 ± 0.07	0.33 ± 0.05	0.42 ± 0.06	r₍rb₎ = -0.682 (-0.927, -0.364)	**0.002**
Node Strength (before)	15.69 ± 3.41	14.57 ± 2.40	17.72 ± 4.11	NS	0.055
Assortativity (before)	– 0.17 ± 0.15	– 0.12 ± 0.11	-0.25 ± 0.19	r₍rb₎ = 0.509 (0.027, 0.927)	**0.022**
Global Efficiency (after)	0.52 ± 0.05	0.54 ± 0.03	0.49 ± 0.06	r₍rb₎ = 0.518 (0.127, 0.855)	**0.020**
Local Efficiency (after)	0.53 ± 0.07	0.52 ± 0.05	0.56 ± 0.08	NS	0.095
Clustering Coefficient (after)	0.34 ± 0.07	0.32 ± 0.05	0.38 ± 0.08	r₍rb₎ = -0.545 (-0.891, -0.118)	**0.014**
Node Strength (after)	16.77 ± 3.12	15.68 ± 2.55	18.77 ± 3.18	r₍rb₎ = -0.527 (-0.864, -0.118)	**0.018**
Assortativity (after)	– 0.17 ± 0.16	– 0.10 ± 0.14	-0.31 ± 0.12	r₍rb₎ = 0.736 (0.427, 0.945)	**< 0.001**
Variables not included in clustering					
Errors	3.71 ± 2.92	3.95 ± 2.96	3.27 ± 2.94	NS	0.464
WM1	16.90 ± 7.74	16.20 ± 7.94	18.18 ± 7.56	NS	0.482
WM2	12.87 ± 6.99	10.80 ± 6.19	16.64 ± 7.05	r₍rb₎ = -0.445 (-0.782, -0.027)	**0.045**
WM3	8.77 ± 5.52	7.50 ± 5.36	11.09 ± 5.28	NS	0.075
WM time 1(minutes)	3.12 ± 1.93	3.21 ± 2.10	2.95 ± 1.66	NS	0.918
WM time 2(minutes)	2.00 ± 1.29	1.73 ± 1.13	2.48 ± 1.48	NS	0.180
WM time 3(minutes)	1.30 ± 0.92	1.13 ± 0.87	1.61 ± 0.97	NS	0.132
Child weekday use of digital devices^d^	0.58 ± 0.72	0.60 ± 0.75	0.55 ± 0.69	NS	0.908
Child weekend use of digital devices^d^	1.19 ± 0.95	1.20 ± 1.01	1.18 ± 0.87	NS	0.965
Сhild age	6.10 ± 1.35	6.00 ± 1.59	6.27 ± 0.79	NS	0.946
Parent age	36.65 ± 5.09	36.10 ± 4.95	37.64 ± 5.43	NS	0.694
Parent weekday use of digital devices^d^	2.29 ± 0.94	2.35 ± 0.93	2.18 ± 0.98	NS	0.631
Parent weekend use of digital devices^d^	1.68 ± 0.87	1.65 ± 0.88	1.73 ± 0.90	NS	0.743
Own device	yes − 14, no − 17	yes − 10, no − 10	yes − 4, no − 7	NS	0.707
Child sex (% girls)	11 (36)	4 (20)	7 (64)	phi = 0.436 (0.071, 0.744)	**0.023**
Played before	yes − 15, no − 16	yes − 10, no − 10	yes − 5, no − 6	NS	0.100
Parent education	Higher − 21, Secondary − 10	Higher − 14, Secondary − 6	Higher − 7, Secondary − 4	NS	0.100

^a^Mean ± SD; n (%).

^b^Mann–Whitney U test, with the effect size reported as the rank-biserial correlation (r₍rb₎, 95% CI); Fisher’s exact test with the effect size φ, 95% CI.

^с^Mann–Whitney U test; Fisher’s exact test.

^d^Ordinal coding: 0 = < 1 h/day; 1 = 1–3 h/day; 2 = 3–5 h/day; 3 = > 5 h/day.

CI - confidence interval; NS - not significant, *p* ≥ 0.05; WM - working memory task score; r₍rb₎ - rank-biserial correlation; phi - phi coefficient.

According to Table 1, graph-theoretical metrics demonstrated significant differences between the two identified clusters in the delta frequency range. Specifically, children from Cluster 1 showed higher global efficiency (before) compared to Cluster 2 (*p* = 0.011), whereas local efficiency (before) and clustering coefficient (before) were significantly higher in Cluster 2 (*p* = 0.011 and *p* = 0.002, respectively). Additionally, assortativity (before) was higher in Cluster 1 (*p* = 0.022). Following the smartphone-based game, global efficiency (after) remained higher in Cluster 1 (*p* = 0.020), while clustering coefficient (after) and node strength (after) were significantly higher in Cluster 2 (*p* = 0.014 and *p* = 0.018, respectively). Moreover, assortativity (after) increased in Cluster 1 compared with Cluster 2 (*p* < 0.001).

Among the non-clustering variables, a significant difference was observed in the second working memory trial (WM2), with higher scores in Cluster 2 (*p* = 0.045). In the categorical variables, a significant association was found between child sex and cluster membership (phi = 0.436, *p* = 0.023), with a higher proportion of girls in Cluster 2.

Subsequently, network metrics were analyzed within the beta frequency range using principal component analysis (PCA) to reduce dimensionality. The sampling adequacy was acceptable (KMO = 0.599; Bartlett’s test: χ² = 211.75, *p* < 0.001). Two principal components were retained for further analyses, together explaining 58.9% of the total variance, with PC1 accounting for 37.2% and PC2 for 21.7% (Supplementary Table 3). PC1 loaded positively on local efficiency and clustering coefficients both before and after the task, but negatively on assortativity. PC2 contrasted node strength with efficiency, loading positively on pre- and post-task node strength and negatively on global efficiency. Higher PC2 scores correspond to denser but less globally efficient network configurations (Supplementary Table 4). These two principal components were subsequently employed for downstream hierarchical clustering analysis.

**Fig. 2:**
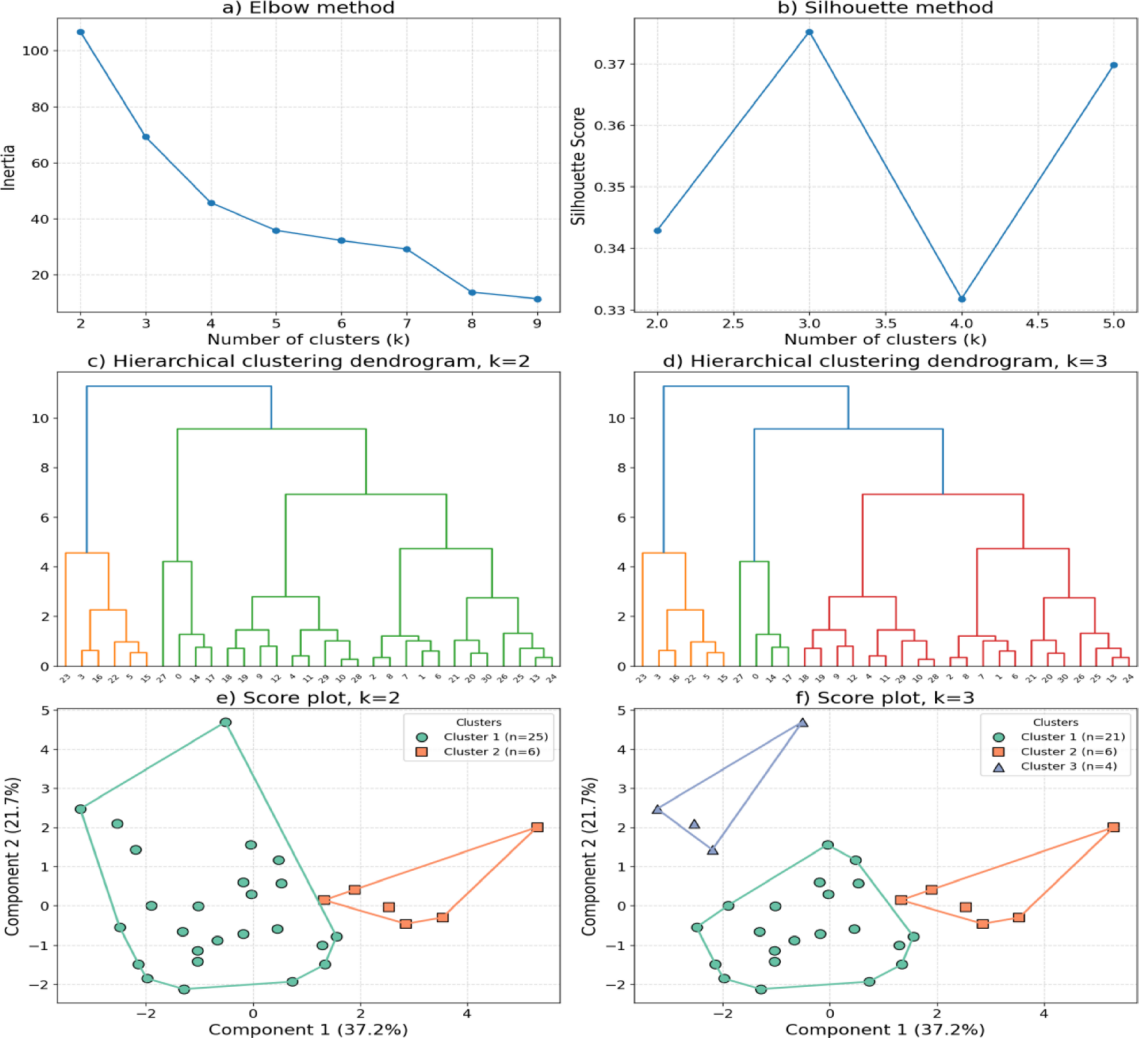
Hierarchical cluster analysis of connectivity metrics in the beta bend. The score plot shows two distinct participant clusters based on the first two principal components.

Both two- and three-cluster solutions were evaluated using hierarchical clustering with Ward’s linkage (Fig. 2). The Elbow plot showed a gradual decrease in within-cluster inertia without a clear inflection point, while the Silhouette coefficient peaked at k = 3. Nevertheless, the three-cluster solution captured only four observations in the smallest cluster, limiting its interpretability in the context of this exploratory study. To preserve a more balanced structure and emphasize the global organization of the data, the two-cluster solution was retained for downstream analysis. Bootstrap-based stability testing confirmed moderate reproducibility for the two-cluster configuration (Jaccard indices: Cluster 1 = 0.741 ± 0.162, Cluster 2 = 0.522 ± 0.243). For comparison, the three-cluster solution yielded higher stability only for one subgroup (Cluster 3 = 0.847 ± 0.288), whereas the others were less consistent (Cluster 1 = 0.659 ± 0.190, Cluster 2 = 0.541 ± 0.232).

As shown in the component score plots (Fig. 2, panels e–f), the two-cluster structure revealed a clear partition of observations along the first two principal components (PC1 = 37.2%, PC2 = 21.7%). Cluster 1 encompassed most subjects, displaying greater dispersion across both components, whereas Cluster 2 formed a smaller, denser group positioned toward higher PC1 values. The overall pattern suggests distinct network organization profiles within the beta band, reflecting potential variability in connectivity dynamics.

**Table 2:** Characteristics of variables included and not included in the clustering analysis in the beta band: differences between the clusters.

Variable	All (*N* = 31)^a^	Cluster 1 (*N* = 25) ^a^	Cluster 2 (*N* = 6) ^a^	Effect size (95% CI) ^b^	*p*-value ^c^
Clustering variables					
Global Efficiency (before)	0.54 ± 0.03	0.54 ± 0.04	0.55 ± 0.02	NS	0.478
Local Efficiency (before)	0.58 ± 0.09	0.55 ± 0.07	0.71 ± 0.06	r_rb = 0.907 (0.693, 1.000)	**< 0.001**
Clustering Coefficient (before)	0.40 ± 0.11	0.37 ± 0.08	0.56 ± 0.10	r_rb = 0.867 (0.600, 1.000)	**< 0.001**
Node Strength (before)	14.96 ± 2.58	14.53 ± 2.38	16.79 ± 2.79	r_rb = 0.640 (0.293, 0.893)	**0.015**
Assortativity (before)	-0.20 ± 0.15	-0.16 ± 0.15	-0.34 ± 0.10	r_rb = -0.733 (-0.933, -0.427)	**0.004**
Global Efficiency (after)	0.55 ± 0.02	0.55 ± 0.03	0.56 ± 0.01	NS	0.190
Local Efficiency (after)	0.59 ± 0.07	0.57 ± 0.06	0.67 ± 0.05	r_rb = 0.827 (0.586, 1.000)	**< 0.001**
Clustering Coefficient (after)	0.40 ± 0.08	0.37 ± 0.06	0.50 ± 0.04	r_rb = 0.933 (0.760, 1.000)	**< 0.001**
Node Strength (after)	15.42 ± 2.55	15.50 ± 2.70	15.10 ± 1.95	NS	0.942
Assortativity (after)	-0.18 ± 0.14	-0.17 ± 0.15	-0.22 ± 0.10	NS	0.608
Variables not included in clustering					
Errors	3.71 ± 2.92	4.08 ± 3.08	2.17 ± 1.47	NS	0.196
WM1	16.90 ± 7.74	17.28 ± 8.11	15.33 ± 6.38	NS	0.565
WM2	12.87 ± 6.99	11.56 ± 6.66	18.33 ± 5.99	r_rb = 0.553 (0.107, 0.920)	**0.040**
WM3	8.77 ± 5.52	7.40 ± 4.93	14.50 ± 4.18	r_rb = 0.727 (0.387, 0.960)	**0.007**
WM time 1(minutes)	3.12 ± 1.93	3.25 ± 2.05	2.59 ± 1.32	NS	0.608
WM time 2(minutes)	2.00 ± 1.29	1.80 ± 1.17	2.81 ± 1.57	NS	0.130
WM time 3(minutes)	1.30 ± 0.92	1.11 ± 0.89	2.11 ± 0.52	r_rb = 0.707 (0.400, 0.933)	**0.006**
Child weekday use of digital devices ^**d**^	0.58 ± 0.72	0.64 ± 0.76	0.33 ± 0.52	NS	0.383
Child weekend use of digital devices ^**d**^	1.19 ± 0.95	1.24 ± 1.01	1.00 ± 0.63	NS	0.727
Сhild age	6.10 ± 1.35	5.96 ± 1.46	6.67 ± 0.52	NS	0.147
Parent age	36.65 ± 5.09	36.52 ± 5.12	37.17 ± 5.42	NS	0.900
Parent weekday use of digital devices ^**d**^	2.29 ± 0.94	2.32 ± 0.95	2.17 ± 0.98	NS	0.683
Parent weekend use of digital devices ^**d**^	1.68 ± 0.87	1.72 ± 0.94	1.50 ± 0.55	NS	0.596
Own device	yes − 14, no − 17	yes − 12, no − 13	yes − 2, no − 4	NS	0.664
Child sex (% girls)	11 (36)	8 (32)	3 (50)	NS	0.638
Played before	yes − 15, no − 16	yes − 11, no − 14	yes − 4, no − 2	NS	0.394
Parent education	Higher − 21, Secondary − 10	Higher − 19, Secondary − 6	Higher − 2, Secondary − 4	NS	0.067

^a^Mean ± SD; n (%).

^b^Mann–Whitney U test, with the effect size reported as the rank-biserial correlation (r₍rb₎, 95% CI); Fisher’s exact test with the effect size φ, 95% CI.

^с^Mann–Whitney U test; Fisher’s exact test.

^d^Ordinal coding: 0 = < 1 h/day; 1 = 1–3 h/day; 2 = 3–5 h/day; 3 = > 5 h/day.

CI - confidence interval; NS - not significant, *p* ≥ 0.05; WM - working memory task score; r₍rb₎ - rank-biserial correlation; phi - phi coefficient.

According to Table 2, significant differences between the two identified clusters were observed in several EEG graph metrics within the beta frequency range. Participants in Cluster 2 demonstrated higher local efficiency (before) (*p* < 0.001), clustering coefficient (before) (*p* < 0.001), and node strength (before) (*p* = 0.015), suggesting a more segregated and densely interconnected functional network structure prior to the task. In contrast, assortativity (before) was significantly lower in Cluster 2 (*p* = 0.004), indicating a weaker tendency of nodes to connect with others of similar degree, a pattern that may reflect reduced network resilience or a less hierarchically organized topology. After the smartphone-based game, the differences in local efficiency (*p* < 0.001) and clustering coefficient (*p* < 0.001) remained significant, again with higher values in Cluster 2.

Among cognitive measures not included in the clustering procedure, participants in Cluster 2 showed higher performance in the second (*p* = 0.040) and third (*p* = 0.007) working memory trials, as well as longer completion time on the third trial (*p* = 0.006). No significant differences were found between the clusters in demographic or digital device use variables.

To evaluate the consistency of clustering solutions obtained separately for the delta and beta frequency bands, a contingency table (Table 3) was constructed to examine the overlap in participant membership between the two sets of clusters.

**Table 3:** Contingency table showing overlap in participant cluster membership between delta and beta frequency bands.

Delta\Beta	β-cluster 1	β-cluster 2
Δ-cluster 1	18	2
Δ-cluster 2	7	4

According to Table 3, eighteen participants (Δ1-β1 = 18) were assigned to the same cluster in both the delta and beta frequency bands, indicating strong consistency in their functional network characteristics across frequencies. In contrast, two participants (Δ1-β2 = 2) belonged to Δ-cluster 1 but shifted to β-cluster 2, suggesting a change in cluster membership between bands. Similarly, seven participants (Δ2-β1 = 7) were part of Δ-cluster 2 but grouped into β-cluster 1, reflecting a reorganization of clustering structure across frequency ranges. Finally, four participants (Δ2-β2 = 4) remained in the same group across both delta and beta bands, showing partial agreement between the two clustering solutions.

To further quantify the degree of overlap, two standard similarity indices were calculated. The Rand Index (RI = 0.574) indicated that approximately 57% of all participant pairs were clustered consistently across the two frequency bands. However, the Adjusted Rand Index (ARI = 0.132), which corrects for chance-level agreement, revealed a relatively low level of consistency between the delta and beta clustering structures.

While the overall correspondence between clustering solutions was modest, the finding that 22 out of 31 participants remained in the same cluster across both delta and beta frequency bands suggests that the overall organization of functional connectivity patterns may be similar between these frequency ranges. However, given the relatively low ARI value, this observation should be considered a preliminary trend rather than conclusive evidence. With a larger sample size, more stable and interpretable cross-frequency clustering patterns may emerge, providing further insight into network organization.

## Discussion

No significant differences were found in EEG functional connectivity metrics across electrode space before and after smartphone gameplay, suggesting that short-term exposure did not alter large-scale network organization. However, cluster analysis revealed distinct network profiles across several frequency bands, which differed in theoretical graph metrics and working memory performance, but not in parameters related to smartphone gameplay or measures of children’s and parents’ digital experience.

Our findings suggest that higher working-memory capacity may be associated with reduced large-scale integration and enhanced local segregation of EEG connectivity network within the delta band, characterized by lower global efficiency together with elevated local efficiency and clustering coefficient values. These results may reflect broader developmental trends, as previous EEG studies have demonstrated a maturational shift in brain oscillations from lower to higher frequency ranges^42^. In line with this transition, younger children rely more on slow oscillatory synchronization to support working-memory engagement, whereas the developmental decrease in delta-theta activity reflects the emerging efficiency of attentional control and processing-speed mechanisms^43^. This could account for the significant effects observed in the low-frequency rhythm, such as the delta band. However, fewer studies have examined functional connectivity in the delta band in relation to cognitive control or working memory.

The delta band is generally associated with deep sleep and relaxation. However, during cognitive engagement, enhanced frontal delta activity has been linked to the suppression of external interference and the facilitation of internally directed attention, potentially through the top-down regulation of task-irrelevant networks^44^.

One review summarized findings on the relationship between working memory, inhibitory control, and delta-band EEG activity. The authors reported that, in adults, delta-band power increased markedly during the maintenance phase of the working memory task, particularly in prefrontal, anterior temporal, and cingulate regions. This enhancement was interpreted as reflecting mature mechanisms of inhibitory control and sustained internal attention, both essential for maintaining information in working memory. In contrast, children aged 8–10 years showed no comparable increase in delta power. Instead, the authors observed enhanced theta-band activity across much of the cortex and a concurrent decrease in alpha power. This pattern was interpreted as evidence that frontal inhibitory systems remain immature at this developmental stage, with children relying more heavily on theta oscillations to support attention and memory maintenance^45^.

Another study in adults demonstrated that delta-band activity carried the most informative signal for distinguishing between different levels of working-memory load, the quantity of information temporarily held and manipulated during task performance. This finding underscores delta activity as a key neurophysiological marker of cognitive engagement, indexing the degree of mental effort and attentional regulation required to sustain working-memory operations^46^. Other research suggests that in early childhood, elevated delta-band activity reflects an excess of broadly distributed, unspecialized cortical connections, which gradually decline as neural circuitry becomes more selective and efficient with age^47^.

Thus, summarizing the findings above, delta rhythm associated with cognitive load may reflect both neural immaturity in children and a mechanism supporting the maintenance of information in working memory. Our results extend this understanding by providing evidence from EEG functional connectivity, showing that a more clustered and less integrated network in the delta band is associated with higher working memory performance. On the other hand, the observed differences between clusters in working-memory performance may also be influenced by sex-related factors, as the cluster demonstrating superior task performance included a significantly higher proportion of girls.

Our findings also reveal a significant difference between clusters in the beta frequency range. Specifically, the cluster demonstrating superior working memory performance is characterized by stronger node strength and higher network clustering, but exhibits lower assortativity within the functional EEG connectivity network.

Beta oscillations have been consistently reported across numerous brain recording studies during perceptual, cognitive, and motor processes^48^. However, the literature on beta-band activity presents mixed evidence regarding functional connectivity during the performance of cognitive tasks. One study reports that the functional network in the beta range shows the most pronounced task-related dynamics during cognitive engagement. As task complexity and cognitive load rise, the dynamic clustering coefficient appears to capture a shift from a predominantly local, segregated network structure toward a more globally integrated organization. These findings imply that, under increased cognitive demands, beta-band connectivity plays a key role in enabling large-scale neural coordination and promoting efficient information transfer across distributed brain regions^49^. Conversely, another study demonstrates that in the beta band, higher values of the clustering coefficient, entropy, and transitivity are observed during problem solving, suggesting a tendency toward increased network segregation under specific cognitive conditions^50^.

Further evidence demonstrates that resting-state EEG-based functional networks in the healthy human brain undergo significant increases in both segregation and integration from early childhood through adolescence, highlighting a developmental refinement of large-scale neural organization^51^. Also, resting-state beta-band mediated networks showed a linear age-related increase in clustering, indicating greater local segregation with cortical maturation^52^.

Accordingly, the observed increase in network clustering (local efficiency and clustering coefficient) associated with higher working memory performance in our study may reflect greater neural maturity, as participants within this cluster were on average older, although the age difference did not reach statistical significance.

Some limitations should be pointed out. The most important limitation of this study is the small sample size of children, which also results in reduced statistical power. We did not apply corrections for multiple comparisons because of the exploratory nature of the analysis. To reduce data dimensionality prior to clustering, we applied principal component analysis jointly to pre- and post-gaming EEG graph metrics. This approach allowed us to capture the shared structure of variation across all measures and participants, rather than timepoint-specific effects. However, combining both timepoints in the same PCA may have introduced mixed time-related variance, potentially reducing the stability of the components and contributing to overfitting in the subsequent clustering analysis.

In conclusion, short-term smartphone gameplay did not produce measurable changes in EEG functional connectivity. Cluster analysis, however, revealed distinct connectivity profiles across frequency bands. Higher working-memory performance was associated with a more locally segregated and less globally integrated delta-band network, as well as with sex-related factors, with a higher proportion of girls in this group. In the beta range, better-performing children exhibited greater local clustering and higher node strength, indicative of more efficient information processing within a more clustered network architecture.

## Methods

### Participants

A total of 58 children initially participated in the study. Data from 27 participants were subsequently excluded due to incomplete data across various assessments (primarily stemming from unreturned parental questionnaires). Consequently, the final analysis included data from 31 children (11 girls), with a mean age of 6.01 ± 1.35 years. Of these, 14 children possessed a personal smartphone.

Parents completed a comprehensive questionnaire, which elicited information regarding parental age, their own screen time on weekdays and weekends, as well as the duration of digital device use by their children acrosscomparable timeframes.

The Ethics Committee at Herzen State Pedagogical University of Russia approved the experimental research protocol (IRB00011060, protocol No. 24, dated November 27, 2023). Written informed consent was obtained from all participants and their legal guardians. The study was conducted in accordance with the ethical principles outlined in the Declaration of Helsinki.

### Study design

The study design involved electroencephalographic (EEG) recordings during two-minute resting-state periods with eyes closed. These recordings were performed in a quiet, isolated room in the morning, both prior to and following a brief smartphone-based gaming session lasting approximately four minutes. This short duration was chosen to capture the acute effects of gameplay while minimizing fatigue in preschool-aged children, who underwent electrode placement, two EEG recordings, and gameplay within a single session.

The mobile game employed is called Talking Tom Gold Run, wherein the cat Tom chases a raccoon who has stolen gold, dodging various obstacles (e.g., cars, boxes). Each collision with an obstacle was counted as an error and recorded for subsequent analysis. The game reset after each collision. Participants controlled the character via touchscreen gestures (e.g., swiping right to execute a turn right). Fifteen of the 31 children had prior experience with this game. The clusters derived from the hierarchical analysis were examined for significant differences based on prior familiarity with the game. Following gameplay and EEG recording, the child rested for 30 min and subsequently performed a working memory task.

### Working memory task

Working memory was evaluated using a computer-based visual working memory task developed by Razumnikova and Nikolaeva^37^. The paradigm consisted of three series, each beginning with the presentation of three familiar visual objects (e.g., flowers, mushrooms, insects) displayed simultaneously on the screen. Participants selected one object by clicking on it with a computer mouse. After each correct selection, a new set was presented in which the number of objects increased by one. On each step, participants were instructed to choose an object that had not been selected previously within the current series. The sequence continued until a repeated selection occurred or the display was filled. Reaction time was not limited. Working memory capacity was quantified as the number of correctly identified new stimuli before the first error. A detailed description of the task is provided by Dydenkova et al. (2024)^53^.

### EEG data acquisition and preprocessing

EEG recordings were acquired using the BE Plus LTM amplifier system (EBNeuro, Italy) with 62 electrodes. The sampling rate was 256 Hz, with each recording lasting two minutes after the cleaning process. Initial preprocessing was performed using EEGLAB (MATLAB‑based). A band‑pass filter ranging from 0.5 to 45 Hz was applied. Artifacts were first manually removed through visual inspection of the EEG recordings. Independent component analysis (ICA) was then performed using the SOBI algorithm implemented in EEGLAB. No more than 10% of the components per recording were identified and removed based on visual evaluation^54^.

Further preprocessing and analysis were performed in Python using the MNE library. Continuous EEG recordings were segmented into non-overlapping 4-second epochs. Functional connectivity between electrode pairs was estimated using the weighted Phase Lag Index (wPLI). For each participant and condition, the resulting wPLI connectivity matrices were averaged within the specified frequency band.

Because functional connectivity matrices are inherently weighted and may include weak or spurious connections, binarization is a commonly used approach to convert these weighted networks into binary ones, enabling a more robust analysis of network topology^55^. Following this approach, a density-based threshold of 0.2 was applied to the weighted connectivity matrices, such that only the top 20% of the strongest wPLI values were retained and set to 1, while the remaining subthreshold connections were set to 0, thereby converting the weighted connectivity matrices into binary adjacency matrices^56^. This threshold was selected based on prior literature and verified through inspection of network density^57,58^. Applying a fixed-density threshold ensured comparability of network topology across participants while retaining the most reliable functional connections.

Connectivity analyses specifically focused on delta (< 4 Hz) and beta (13–30 Hz) frequency bands. Graph‑theoretical metrics, namely: global and local efficiency, clustering coefficient, node strength, and assortativity, were calculated using built‑in functions of the NetworkX Python package. Node strength was defined as the mean sum of edge weights per node, computed on the weighted functional connectivity matrix prior to thresholding. All other network measures were calculated on the binarized connectivity matrix obtained after applying a proportional threshold of 0.2.

### Dimensionality reduction and clustering

All variables were standardized via z‑score transformation, thereby bringing diverse measurement scales to a common scale and mitigating scale‑related biases^59^. To reduce data dimensionality prior to clustering, principal component analysis (PCA) was applied jointly to pre- and post-gaming EEG graph metrics to capture the shared structure of variation across all measures and participants, rather than timepoint-specific effects. Sampling adequacy was verified using the Kaiser‑Meyer‑Olkin (KMO) measure and Bartlett’s test of sphericity. The first two principal components, which accounted for the largest portion of variance, were extracted for subsequent analyses.

A hierarchical cluster analysis was performed using Ward’s method and Euclidean distance^60^. The optimal number of clusters was determined by applying the elbow method, silhouette analysis and visual inspection of the dendrogram. Cluster stability was assessed using the mean Jaccard coefficient. Values above 0.75 were considered to indicate stable clusters, coefficients between 0.60 and 0.75 were interpreted as reflecting consistent patterns in the data, values between 0.50 and 0.60 indicated unstable clusters, and those below 0.50 were classified as dissolved^61^. All computations were conducted in Python using Scikit‑learn^62^. The resulting clusters were visualized in PCA‑reduced component space. To examine differences between clusters, pairwise comparisons were conducted using the Mann–Whitney U test for continuous variables and Fisher’s exact test for categorical variables. Effect sizes were reported as rank-biserial correlation and phi coefficients, respectively. The statistical significance threshold was set at *p* < 0.05.

All visualizations were generated using Matplotlib (version 3.10.0, https://matplotlib.org), NumPy (version 2.0.2, https://numpy.org), and Scikit‑learn (version 1.6.1, https://scikit-learn.org).

## Supplementary Information

Below is the link to the electronic supplementary material.

Supplementary Material 1

## Data Availability

The datasets analysed during the current study are not publicly available due to governing Russian data protection laws but are available from the corresponding author upon reasonable request in anonymized form.
