# Supplementary material for: Functional connectivity patterns in preschool children associated with working memory performance and digital device use
Source: Sci Rep. 2025 Dec 30;16:1308. doi: 10.1038/s41598-025-33555-w (PMC12796410; doi:10.1038/s41598-025-33555-w)
Supplement: Supplementary file 1 — Supplementary Material 1 [file 41598_2025_33555_MOESM1_ESM.docx]

Supplementary File 1. Tables related to the PCA analysis

| **Component** | **Explained Variance (%)** | **Cumulative Variance (%)** |
| --- | --- | --- |
| PC1 | 34 | 34 |
| PC2 | 22,77 | 56,77 |
| PC3 | 15,33 | 72,1 |
| PC4 | 8,5 | 80,6 |
| PC5 | 7,07 | 87,67 |
| PC6 | 5,01 | 92,69 |
| PC7 | 3,71 | 96,39 |
| PC8 | 2,93 | 99,33 |
| PC9 | 0,39 | 99,71 |
| PC10 | 0,29 | 100 |

Supplementary table 1. Variance explained by principal components (delta band). PC: principal component.

| **Variables** | **PC1** | **PC2** |
| --- | --- | --- |
| Global Efficiency (before) | -0,296 | -0,079 |
| Local Efficiency (before) | 0,334 | -0,245 |
| Clustering Coefficient (before) | 0,385 | -0,26 |
| Node Strength (before) | 0,352 | 0,183 |
| Assortativity (before) | -0,308 | -0,242 |
| Global Efficiency (after) | -0,114 | 0,512 |
| Local Efficiency (after) | 0,362 | 0,391 |
| Clustering Coefficient (after) | 0,416 | 0,282 |
| Node Strength (after) | 0,25 | -0,403 |
| Assortativity (after) | -0,235 | 0,341 |

Supplementary table 2. Principal component analysis loadings for delta band. Before: before the game; After: after the game.

| **Component** | **Explained Variance (%)** | **Cumulative Variance (%)** |
| --- | --- | --- |
| PC1 | 37,16 | 37,16 |
| PC2 | 21,71 | 58,87 |
| PC3 | 12,49 | 71,36 |
| PC4 | 8,07 | 79,43 |
| PC5 | 6,54 | 85,97 |
| PC6 | 6,13 | 92,1 |
| PC7 | 4,69 | 96,79 |
| PC8 | 2,68 | 99,48 |
| PC9 | 0,29 | 99,77 |
| PC10 | 0,23 | 100 |

Supplementary table 3. Variance explained by principal components (beta band). PC: principal component.

| **Variables** | **PC1** | **PC2** |
| --- | --- | --- |
| Global Efficiency (before) | 0,116 | -0,464 |
| Local Efficiency (before) | 0,462 | -0,029 |
| Clustering Coefficient (before) | 0,454 | 0,067 |
| Node Strength (before) | -0,302 | -0,16 |
| Assortativity (before) | 0,179 | -0,416 |
| Global Efficiency (after) | 0,409 | -0,031 |
| Local Efficiency (after) | 0,423 | 0,103 |
| Clustering Coefficient (after) | -0,276 | -0,133 |
| Node Strength (after) | 0,104 | 0,509 |
| Assortativity (after) | -0,106 | 0,541 |

Supplementary table  4. Principal component analysis loadings for beta band. Before: before the game; After: after the game.
